# Important Variables When Screening for Students at Suicidal Risk: Findings from the French Cohort of the SEYLE Study

**DOI:** 10.3390/ijerph121012277

**Published:** 2015-09-30

**Authors:** Jean-Pierre Kahn, Alexandra Tubiana, Renaud F. Cohen, Vladimir Carli, Camilla Wasserman, Christina Hoven, Marco Sarchiapone, Danuta Wasserman

**Affiliations:** 1 Centre Hospitalier Universitaire de Nancy, Hôpitaux de Brabois, Vandoeuvre les Nancy 54511, France; 2 Université de Lorraine, Faculté de Médecine, Vandoeuvre les Nancy 54505, France; 3 Centre Psychothérapique de Nancy, Laxou 54520, France; E-Mails: alexandra.tubiana@cpn-laxou.com (A.T.); r.cohen@chu-nancy.fr (R.F.C.); 4 National Centre for Suicide Research and Prevention of Mental lll-Health (NASP)/WHO Collaborating Centre for Research, Methods Development and Training in Suicide Prevention, Karolinska Institute, Stockholm SE-171 77, Sweden; E-Mails: vladimir.carli@ki.se (V.C.); Danuta.Wasserman@ki.se (D.W.); 5 Department of Child and Adolescent Psychiatry, New York State Psychiatric Institute, Columbia University, New York, NY 10032, USA; E-Mails: camillawasserman@gmail.com (C.W.); HOVEN@nyspi.columbia.edu (C.H.); 6 Department of Health Sciences, University of Molise, Campobasso 86100, Italy; E-Mail: marco.sarchiapone@gmail.com

**Keywords:** suicide, prevention, psychopathology, professional screening, two-stage screening, adolescents, French SEYLE cohort, SEYLE

## Abstract

Due to early detection of mental ill-health being an important suicide preventive strategy, the multi-centre EU funded “Saving and Empowering Young Lives in Europe” (SEYLE) study compared three school-based mental health promotion programs to a control group. In France, 1007 students with a mean age of 15.2 years were recruited from 20 randomly assigned schools. This paper explores the French results of the SEYLE’s two-stage screening program (ProfScreen) and of the cross-program suicidal emergency procedure. Two-hundred-thirty-five ProfScreen students were screened using 13 psychopathological and risk behaviour scales. Students considered at risk because of a positive finding on one or more scales were offered a clinical interview and, if necessary, referred for treatment. A procedure for suicidal students (*emergency* cases) was set up to detect emergencies in the whole cohort (*n* = 1007). *Emergency* cases were offered the same clinical interview as the ProfScreen students. The interviewers documented their reasons for referrals in a short report. 16,2% of the ProfScreen students (38/235) were referred to treatment and 2,7% of the *emergency* cases (27/1007) were also referred to treatment due to high suicidal risk. Frequent symptoms in those students referred for evaluation were depression, alcohol misuse, non-suicidal self-injuries (NSSI), and suicidal behaviours. According to the multivariate regression analysis of ProfScreen, the results show that the best predictors for treatment referral were NSSI (OR 2.85), alcohol misuse (OR 2.80), and depressive symptoms (OR 1.13). Analysis of the proportion for each scale of students referred to treatment showed that poor social relationships (60%), anxiety (50%), and suicidal behaviours (50%) generated the highest rate of referrals. Qualitative analysis of clinician’s motivations to refer a student to mental health services revealed that depressive symptoms (51%), anxiety (38%), suicidal behaviours (40%), and negative life events (35%) were the main reasons for referrals. Thus, not only the classical psychopathological symptoms, such as depression, anxiety, and suicidal behaviours, but also negative life events and poor social relationships (especially isolation) motivate referrals for treatment.

## 1. Introduction

Although there has been a general decline in global suicide rates between 2000 and 2012 [1], suicide is still a public health concern. It is the second leading cause of death in young people aged 15–29 years worldwide [1,2], and the main cause of death among those aged 15–29 years in high income countries, including France [3].

It has been shown that about 90% of suicidal persons suffer from mental disorders or psychiatric symptoms [4]. Suicide preventive actions should, therefore, take into account the key role of mental disorder management: it has been calculated that if 50% of people suffering from mood disorders, schizophrenia or alcohol-related disorders were efficiently treated, suicides would decrease by 20,5%, saving 165 000 lives per year worldwide [5].

Since the majority of mental disorders in adulthood emerge during adolescence, with an average onset for any mental disorder at 14 years-old [6,7], suicide prevention actions should focus on early detection to avoid aggravation and persistence of emergent mental health problems.

Screening is a method used to identify, in larger populations, those who suffer from a preclinical condition [8]. It is not just a test, but a program involving a sequence of events provided separately or in a coordinated and quality-assured system [9]. One of the oldest screenings of medical history was initiated in the United States army (1917) to detect psychologically ill-suited soldiers [8]. It included a two-stage process, with a first step identifying positive cases, followed by an individual psychological examination of the positive cases that confirmed or rejected true positivity.

The SEYLE (Saving and Empowering Young Lives in Europe) study has compared screening to two other school-based suicide prevention methods and to a control group, in 11 countries [10]. This paper presents: (1) the results of the screening program initiated in the French cohort of the SEYLE study, focusing on the results of the students referred to mental health care services after clinical interview; and (2) the major reasons that led the clinicians to refer students to mental health facilities.

## 2. Material and Method

### 2.1. The SEYLE Study

**Figure 1:**
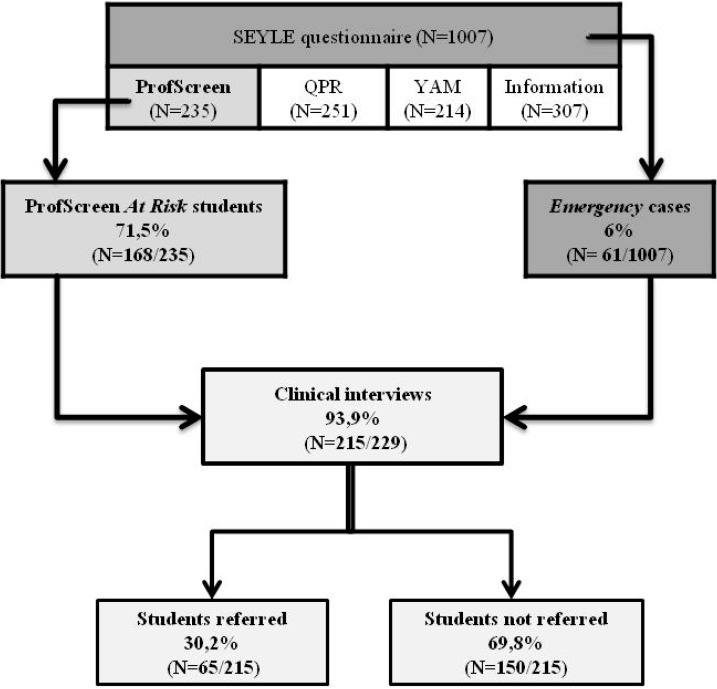
Screening, interview, and referral procedures

The SEYLE study is a multicenter clinical cluster-randomized controlled trial, funded by the Seventh Framework Programme of the European Union (FP7), and performed in 10 European Union countries and Israel. It is coordinated by the National Centre for Suicide Research and Prevention of Mental Ill-Health at Karolinska Institute (Stockholm, Sweden). The SEYLE study is registered at the German Clinical Trials Register (DRKS00000214) for the 10 European countries, and, in Israel, at the US National Institute of Health (NIH) clinical trial registry (NCT00906620). It has received the authorization of the French Ethical Committees and related agencies (Comité de Protection des Personnes Sud-Méditerranée II Marseille 2; Agence Francaise de Sécurité Sanitaire des Produits de Santé; Comité Consultatif sur le Traitement de l’Information en matière de Recherche dans le domaine de la Santé). The detailed protocol has been published elsewhere [10]. The SEYLE study aimed at collecting epidemiological data on European adolescents’ health, well-being, and values using a self-report questionnaire and comparing preventive and mental health promotion programs. The SEYLE study tested and compared three different active programs to a control group (Figure 1):

(1) “Question Persuade and Refer” (QPR) gatekeeper program [11], a training of the adults working in the school;
(2) a mental health awareness program designed for students called the “Youth Aware of Mental Health” (YAM) program [12];
(3) a two-stage health professional screening of *at risk* students “ProfScreen” [13]; and
(4) a minimal information program considered as the control group “Information”.

Participating schools were public, contained at least forty 15-year-old pupils, had more than two teachers for pupils 15 years of age and no more than 60% of the pupils were of the same gender [10]. The four programs were randomly assigned to the schools with only one program per school. All 14 to 16 years-old students of one school were invited to take part in the same program.

All the recruited students (France: median age 15.2; SD 0.8 [14]) filled a 127 item baseline questionnaire. The four programs were implemented in the schools during the month following the questionnaire.

A standardized methodology has been designed by the coordinating center in order to ensure a consistent implementation of the study in all countries [14]. Nevertheless, cultural and academic system variations across participating countries introduced different settings for performing the clinical interviews in the ProfScreen program, e.g., hospitals, local outpatient clinics or in schools, which was the case in France.

The French Ethical Committees required that both parents (or legal guardians) signed the informed consent. This consent stipulated that the student could be contacted for a clinical interview performed on the school premises.

1007 students were recruited in all 20 schools of the SEYLE French site (Lorraine region). Among this group, 235 took part to the ProfScreen program.

### 2.2. Self-Report Questionnaire Screening

#### 2.2.1. The SEYLE Questionnaire

The SEYLE questionnaire (completed in class by all the students of the cohort) explored various aspects of adolescents’ mental health and lifestyles, including sociodemographic data, psychopathology, at risk behaviors, values and interests. The instruments used in the questionnaire were: the Beck Depression Inventory II (BDI-II), minus the item on libido; the Zung Self-Assessment Anxiety Scale (SAS); the Paykel Suicidal Scale (PSS); the Young Diagnostic Questionnaire (YDQ); questions issued from the Deliberate Self-Harm Inventory (DSHI) and the Global School-Based Student Health Survey (GSHS); the WHO Well-being scale (WHO-5), as well as other questions specifically tailored for the SEYLE questionnaire.

#### 2.2.2. ProfScreen: *At Risk* Students

In the schools that were allotted to the ProfScreen program, the SEYLE questionnaire was used to identify *at risk* students. The students reaching the threshold for at least one of the 13 scales established in the SEYLE study protocol [13] were offered a clinical evaluation by a psychiatrist or a clinical psychologist. This interview could either lead, or not lead, to a referral to mental health facilities according to the clinician’s judgment and based on his/her global clinical impression. The scales and cut-offs are described in Table 1.

**Table 1:** Scales and cut-offs used in the ProfScreen arm.

Scales	Cut Offs
Depression (BDI)	≥superior or equal to 14 (Mild depression)
Anxiety (SAS)	≥superior or equal to 45 (Mild anxiety)
Suicidal ideations/behaviors (PSS) + question about previous suicide attempt	any suicidal thought or attempts in the last two weeks a life-time history of suicide attempt
Non-suicidal self-injury (Shortened version of DSHI)	a life-time history of two or more incidents of intentional self-injury
Eating behavior (BMI)	BMI < 16,5
Sensation seeking and delinquent behaviors	three or more positive answers out of six
Tobacco *	≥2 cigarettes per day (France: ≥7)
Alcohol	student has/had: an alcoholic drink at least twice a week and/or drinks at least three alcohol units per occurrence and/or got drunk at least three times (lifetime) and/or at least three hangovers (lifetime)
Illegal drugs	illegal drug used three times or more during lifetime
Excessive use of media	Over five hours per day in front of a screen
Loneliness/Social relationships problems	Student feels lonely most of the time in the past 12 months
Bullying	≥5 incidents (chosen out of 15) in the past 12 months
Truancy	Student missed class 3 times or more in the past two weeks (in France: ≥ 2 because the school year started a week before the program started and we considered necessary to modify this cut off in order to avoid a bias)

* For this scale, cut-offs could be adapted in each country.

### 2.3. Emergency Cases

Since all the students of the study had to fill the SEYLE questionnaire, it was ethically unacceptable not to react to students claiming suicidal ideation/behaviors.

Students’ questionnaires were manually checked by SEYLE staff immediately after they were completed. When a student answered “*Sometimes*”, “*Often*”, “*Very often*”, or “*Always*” to the question: “*During the past two weeks, have you reached the point where you seriously considered taking your life or perhaps made plans on how you would go about doing it*?” and/or “*Yes, during the past two weeks*” to the question “*Have you ever tried to take your own life?*” he/she was offered a clinical interview as soon as possible. This interview was the same as the one offered to at risk students in the ProfScreen program (see Section 2.4).

### 2.4. Interviews and Referrals

Students detected as being *at risk* in the ProfScreen arm were offered the clinical interview within three days. Emergency cases in all four arms of the program were offered the interview as soon as possible. The interview was performed at the school premises (most of the time, in the school nurse office). The parents of the students were not contacted prior to the clinical interview, since they previously agreed to it in signing the informed consent before entering the study. The clinician made a phone report to the parents, after the interview, in the presence of the student, without disclosing any detail. In France, the clinicians were asked to describe explicitly the reason(s) for their referral in a short written report.

The clinical interview is based on a one-hour semi-structured questionnaire (different from the SEYLE questionnaire) exploring: depression, anxiety, suicidal behaviors, non-suicidal self-injuries, risk behaviors, irritability/anger, addictions, truancy, bullying, dietary habits, sexuality, and social relationships. The clinician was aware if the student was interviewed for a suicidal emergency situation or for being *at risk* on at least one of the ProfScreen scales, and then chose to refer the student to mental health facilities, or not, on the basis of his/her own clinical evaluation.

The screening process from the questionnaire to the clinical interview is described in Figure 1.

### 2.5. Statistical Procedures

As described above, *at risk* students in the ProfScreen arm and *emergency* cases in the whole cohort, were identified via the SEYLE questionnaire. Those among them who were then referred were identified via the clinical interview reports. The percentage of students (*at risk* and *emergencies*) meeting the cut-off criteria (threshold scores) of each scale was calculated.

In order to determine the positive predictive value of the different scales, the proportion of those who were referred for treatment was calculated for the students who reached cut-off points for at least one scale.

To test the sensitivity of the different scales, the proportion of students referred or not referred was calculated for each scale.

The clinician’s reasons to refer students have been assessed with an analysis of the clinicians’ reports. Reports have been reviewed manually to calculate the number of referred students and to extract key words that explained the referrals (reasons for referral). Several key words were accepted for the same referral.

All statistical analyses were performed with SAS software package, version 9.1 (SAS Institute Inc., Cary, NC, USA).

## 3. Results

### 3.1. Referrals

Among the 235 students of the ProfScreen cohort, 168 (71,5%) screened positive on one scale or more. Thirty-eight students (16,2%) were referred for treatment by the clinician. *Emergency* cases represented 61 students (6%) out of 1007 participants (in the four programs) in the French cohort. Twenty-seven students (2,7%) were referred for treatment (Table 2).

**Table 2:** Proportion of students referred in the ProfScreen arm and *emergency* cases in Lorraine region (France).

Total Sample	ProfScreen		*Emergency* Cases	
	N = 235		N = 1007	
	N	%	N *	%
Students screened positive	168	71,5	61	6
Students referred for treatment	38	16,2	27	2,7

* Including *Emergency* cases of the ProfScreen arm.

Regarding referrals, there is no statistically significant difference between psychiatrists and psychologists (*p* > 0.10). Psychiatrists referred 17 students out of 53 (32%) and psychologists referred 21 students out of 103 (20,4%).

### 3.2. Psychopathology

As shown in Table 3, the most frequent scales triggered by the ProfScreen students were alcohol misuse (50%), depression (36,3%), non-suicidal self-injuries (35,1%), and bullying (26,2%), while suicidal behaviors ranked fifth (21,4%). It is important to note that, in spite of an early implementation in the academic year (*i.e.*, two weeks after school starts), the truancy scale was triggered by 16,1% of the students. However, sensation seeking has almost never been encountered in the French cohort (0,6%). Media exposure (6,5%) and BMI (7,1%) were also among the scales that were not often triggered. 73,7% of the referred students triggered the depression scale, followed by alcohol misuse (52,6%), non-suicidal self-injury (50%), suicidal behaviors (44,7%), bullying, and anxiety (34,2%) scales. Social relationships ranked as 6th with 23,7% of referrals.

It should be noted that among the 36 students who were detected positive for suicidal behaviors in the ProfScreen arm, 5 (13,9%) did not trigger any other scales.

**Table 3:** Percentage of *At risk* and Referred students per scale.

Scales	*At risk* Students (N = 168)		Referred Students (N = 38)		*p* Value *
	N	%	N	%	
Alcohol	84	50,0	20	52,6	NS
Depression	61	36,3	28	73,7	<0.001
NSSI	59	35,1	19	50,0	0.0525
Bullying	44	26,2	13	34,2	NS
Suicidal behaviours	36	21,4	17	44,7	<0.001
Truancy	27	16,1	6	15,8	NS
Anxiety	26	15,5	13	34,2	<0.001
Illegal drugs	21	12,5	6	15,8	NS
Tobacco	19	11,3	4	10,5	NS
Social relationships	16	9,5	9	23,7	<0.01
Eating habits	12	7,1	1	2,6	NS
Media exposure	11	6,5	4	10,5	NS
Sensation seeking	1	0,6	0	0,0	NS

* Fisher’s test on the difference in the proportion of students reaching the threshold of a scale between at risk and “referred” students.

### 3.3. Scales and Referrals

The multivariate regression performed to find out the best referral predictors (Table 4) showed that the three scales most frequently associated with the decision of clinicians to refer the adolescent to treatment were non-suicidal self-injury (OR: 2.85), alcohol misuse (OR: 2.80) and depressive symptoms (OR: 1.13).

**Table 4:** Scores at scales and prediction at referral (results of the bivariate and stepwise multivariate regressions).

Scales	OR in Bivariate Regression [CI: 95%]	OR in Multivariate Regression [CI: 95%]
Beck Depression Inventory BDI-II	1.15 ** [1.09–1.22]	1.13 ** [1.07–1.20]
Zung Self-Assessment Anxiety Scale SAS	1.11 ** [1.06–1.17]	NS
Non Suicidal Self Injury NSSI	3.10 ** [1.38–6.95]	2.85 * [1.03–7.90]
Alcohol consumption	2.89 ** [1.35–6.22]	2.80 * [1.05–7.43]

** <0.01; * <0.05.

**Figure 2:**
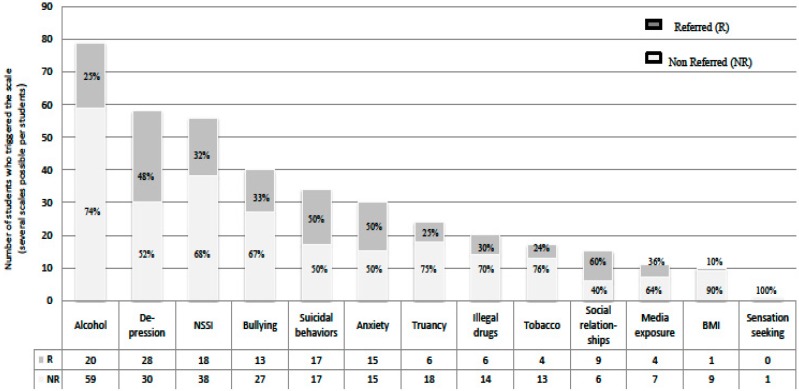
Proportion of referred students for each scale.

An analysis of the proportion of referred students for each scale showed that poor social relationships (60%), anxiety (50%) and suicidal behaviors (50%) were the scales that generated the highest proportion of referred students (Figure 2).

### 3.4. Reasons for Referrals

As described previously, the French clinicians had to produce a short report on which clinical ground(s) they decided to refer students to treatment. The analysis of these clinical features (Table 5) showed that the most frequent reasons to refer a student to mental health care for treatment were depressive symptoms (50,8%), suicidal behaviors (39,7%), anxiety (38,1%), and negative life events (34,9%).

**Table 5:** Clinicians reasons for referrals to mental health care *.

ProfScreen + Emergencies (baseline)	n = 63 **	
	n	%
Depressive symptom	32	50,8
Suicidal behaviours	25	39,7
Anxiety	24	38,1
Negative life events	22	34,9
Non-Suicidal Self-Injury	12	19,0
Irritability	10	15,9
Family conflicts	10	15,9
Eating disorder	6	9,5
Alcohol	6	9,5
Low self esteem	5	7,9
Impulsivity	4	6,3
Tobacco	4	6,3
Sleep Disorder	4	6,3
Drugs	3	4,8
Risk behaviours	3	4,8
Social relationships (isolation)	3	4,8
Behaviour disorder	2	3,2
Bullying	2	3,2
Domestic violence	2	3,2
Sexual harrassement	2	3,2
Parentification	1	1,6
Phobia	1	1,6

* Reasons are cummulative; ** 2 Reports missing.

## 4. Discussion

The goal of clinical screening is to provide an early treatment to people who need it. To achieve this goal, the program must achieve good acceptability with the targeted subjects. It has been reported by Miller *et al.* [15,16], that for school psychologists, school principals, and students, school-based screening programs were the least acceptable of three types of suicide prevention programs (curriculum-based programs presented to students, in-service presentations to school staff, and student self-report screening measures). Our experience contrasts with these results: the high rate of attendance at the clinical interviews (215/229: 93,9%) in the SEYLE-participating schools in France demonstrated that the program has been perceived positively by the students. Many of them have shown interest by asking questions or seeking advice during the clinical interview. During the phone report, parents often expressed their satisfaction in receiving a professional opinion on their child’s mental health (whether it was satisfactory or an indication for treatment) and sometimes contacted us later to get more specific assistance.

### 4.1. False Positive Cases are Functionally Impaired

A total of 16,2% of the ProfScreen students were referred to mental health care after the clinical interview, while 71,5% had been detected *at risk*. As for the *emergency* cases, 61 (6%) students out of 1007 were detected and 27 (2,7%) were referred to treatment. The number of referred students agrees with the 15,2% found after screening and clinical interview by Husky *et al.* [17], even though areas explored in their screening were not exactly the same as in SEYLE.

The large difference between *at risk* students and referred students shows that a large number of false positive cases are generated by the questionnaire-based screening, but higher cut-offs in screening could have increased the number of false negatives, leading to serious life-threatening incidents [18]. Furthermore, as demonstrated by Leon *et al.*’s study, clinical interviews with false positive cases could be of interest for patients since false positives from psychiatric screening have significantly greater functional impairment and higher rates of use of mental health services than the general population [19]. Laukkanen *et al.* have also shown that a brief intervention may be useful and sufficient for adolescents with a low threshold disorder [20]. The second stage (clinical interview) is therefore an important part of the program, even though, it is probably costly and/or time consuming. This first contact with mental health professionals, by initiating early treatment (whether brief or long term), may help to reduce the direct and indirect costs that mental ill-health imposes on the community. A full-scale cost/benefit evaluation should be made but this would require a long term study.

### 4.2. Risk Behavior and Psychopathology

The most salient difference between referred students and *at risk* students is found in the number of scales triggered. Referred students are significantly more depressed (*p* < 0.001), have more suicidal ideation/behavior (*p* < 0.001), are more anxious (*p* < 0.001) and more socially isolated (*p* < 0.01) than non-referred *at risk* students. The ranking of the expected classical symptomatology triptych (depression, anxiety, and suicidality) differs between those referred and non-referred. The present study also shows that although social isolation is an understudied topic, it is an important differentiating factor between referred and non-referred students.

The best predicting scales for referrals are non-suicidal self-injury, alcohol misuse, and depression. Surprisingly, the suicidal behaviors scale was not among the best predicting scales, in spite of the seriousness of the condition. Even though the delay between filling the questionnaire and clinical interview was as short as possible, the fluctuating nature of suicidal ideation may explain why it is not among the best predicting scales. This hypothesis may be supported by the observation that, the longer the ideations persists, the more severe are the thoughts [21]. Interestingly, the scale measuring social relationships appears to be the scale with the highest ratio of referred students.

Suicidal behaviors, anxiety, depression, and media exposure scales also show a high specificity, in contrast to BMI, bullying, and truancy scales. In this cohort, having problematic social relationships (isolation) combined with media exposure reinforces Carli *et al.*’s findings of a group with an “invisible risk for psychopathology and suicidal behaviour” [22]. The invisible group does not score high on all at risk behaviors, but only on media exposure, sedentary behavior and reduced sleep. This “invisible group” shows high prevalence of depression and suicidal behaviors. Therefore, such risk behaviors should not be neglected or disregarded when compared to alcohol/drug abuse, sensation seeking or truancy, because they were shown to be associated with mental distress and psychopathology [22].

### 4.3. Reasons to Refer

The analysis of clinicians’ reasons to refer shows that the classical psychopathological symptoms (depression, anxiety, suicidal behaviors) most frequently explain the referral. Negative life events are also frequently listed as a reason to refer. This aspect should be emphasized, since it has been demonstrated that stressful life events, in particular health- and work-related life events [23], are risk factors and key precipitants of suicide attempts [24]. On the contrary, social isolation was rarely mentioned as a reason for referral despite isolated students being found in high proportions in the referred group. The finding is similar for bullying and truancy, which generated a very low number of referrals.

Finally, 118 ProfScreen students and 32 Emergency students who attended the interview were not referred. A qualitative analysis of the French SEYLE mental health clinicians reports showed that for 32 of them, the clinical interview and the phone report to the parents had been beneficial enough to avoid making an appointment to mental health services [25]. The 118 left were considered as true negatives. All students were informed that the SEYLE team was available for further help anytime in the future.

## 5. Conclusions

In France, the results of the ProfScreen program and the *emergency* screening procedure of the SEYLE study have both shown that screening is an efficient method to refer students in need of treatment. The present study confirms that classical psychopathology symptoms and risk behaviors are good predictors and motivators for clinical referral. The two-stage process highlighted that some less often identified variables, such as negative life events, poor social relationships or media exposure deserve closer attention by clinicians, because those variables are also markers of psychopathology and poor functionality and need to be taken into account when screening for adolescents with suicidal ideations/behaviors or poor mental health risk.
